# Sensory ataxia-plus secondary to cervical spondylotic myelopathy

**DOI:** 10.1055/s-0043-1763300

**Published:** 2023-05-09

**Authors:** Nagyla Aparecida Barros, Luis Eduardo Borges de Macedo Zubko, Igor Abrahim Nascimento, Léo Coutinho, Hélio Afonso Ghizoni Teive

**Affiliations:** 1 Universidade Federal do Paraná, Hospital de Clínicas, Serviço de Neurocirurgia, Curitiba PR, Brazil.; 2 Universidade Federal do Paraná, Hospital de Clínicas, Serviço de Neurologia, Unidade de Distúrbios de Movimento, Curitiba PR, Brazil.; 3 Universidade Federal do Paraná, Hospital das Clínicas, Departamento de Clínica Médica, Programa de Pós-Graduação em Medicina Interna e Ciências da Saúde, Curitiba PR, Brazil.

**Figure 1:**
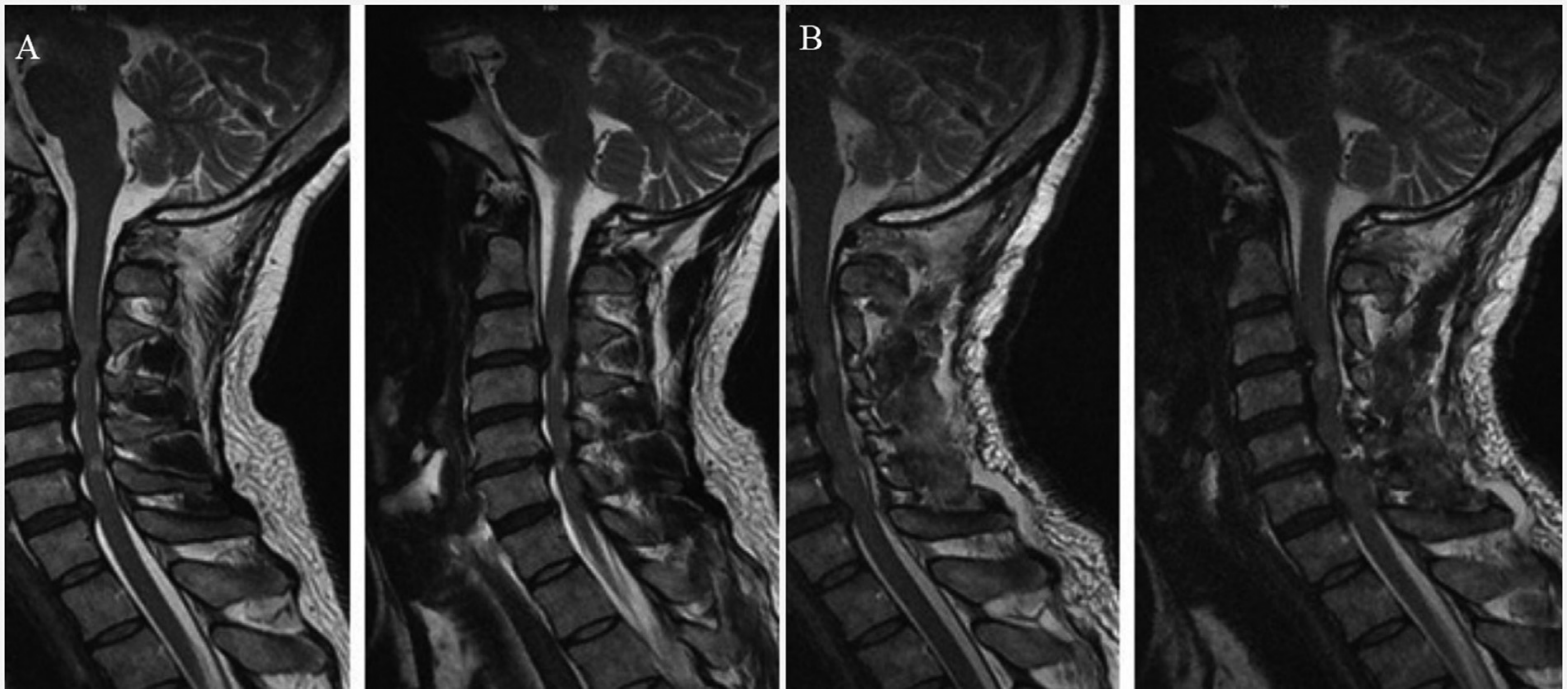
Cervical spinal cord T2-weighted MRI revealing severe degenerative disk disease and compressive spondylotic myelopathy at the levels of C3-C4 and C5-C6 ( **A** ). Cervical spinal cord T2-weighted MRI showing signs of posterior cervical decompression and cervical laminoplasty involving C3-C7 ( **B** ).

A 45-year-old male patient presented a 2-week history of progressive gait imbalance. He also presented impaired proprioception, symmetric distal quadriparesis (grade 4/5), gait ataxia with a positive Romberg sign, bilateral upper limb dysmetria, and dysdiadochokinesia. The patient did not present nystagmus and/or dysarthria. A cervical spine magnetic resonance imaging (MRI) scan revealed severe degenerative disk disease and compressive spondylotic myelopathy at C3-C4 and C5-C6 ( Figure 1A ). He was submitted to posterior decompression and laminoplasty involving C3-C7 ( Figure 1B ), and presented improvement in gait. Mild cerebellar signs in a patient with a positive Romberg sign, without dysarthria and nystagmus, point to a sensory ataxia-plus rather than a cerebellar etiology. 1 2
